# Accurate temperature diagnostics for matter under extreme conditions

**DOI:** 10.1038/s41467-022-35578-7

**Published:** 2022-12-23

**Authors:** Tobias Dornheim, Maximilian Böhme, Dominik Kraus, Tilo Döppner, Thomas R. Preston, Zhandos A. Moldabekov, Jan Vorberger

**Affiliations:** 1 Center for Advanced Systems Understanding (CASUS), Görlitz, D-02826 Germany; 2 Helmholtz-Zentrum Dresden-Rossendorf (HZDR), Dresden, D-01328 Germany; 3 Technische Universität Dresden, Dresden, D-01062 Germany; 4 Institut für Physik, Universität Rostock, Rostock, D-18059 Germany; 5 Lawrence Livermore National Laboratory, Livermore, CA 94550 USA; 6 European XFEL, Schenefeld, D-22869 Germany

**Keywords:** Astrophysical plasmas, Laser-produced plasmas

## Abstract

The experimental investigation of matter under extreme densities and temperatures, as in astrophysical objects and nuclear fusion applications, constitutes one of the most active frontiers at the interface of material science, plasma physics, and engineering. The central obstacle is given by the rigorous interpretation of the experimental results, as even the diagnosis of basic parameters like the temperature *T* is rendered difficult at these extreme conditions. Here, we present a simple, approximation-free method to extract the temperature of arbitrarily complex materials in thermal equilibrium from X-ray Thomson scattering experiments, without the need for any simulations or an explicit deconvolution. Our paradigm can be readily implemented at modern facilities and corresponding experiments will have a profound impact on our understanding of warm dense matter and beyond, and open up a variety of appealing possibilities in the context of thermonuclear fusion, laboratory astrophysics, and related disciplines.

## Abstract (web summary)

Existing methods for temperature estimation of warm dense matter rely on model calculations. Here the authors report a method to extract the temperature of complex materials that is previously only inferred by using model calculations.

## Introduction

The study of matter at extreme conditions (temperatures of *T* ~ 10^4^ − 10^8^K and pressures of *P* ~ 1 − 10^4^Mbar) constitutes one of the most fundamental challenges of our time^1^. Such warm dense matter (WDM)^2,3^ is ubiquitous throughout our Universe^4^ and naturally occurs in a number of astrophysics objects^5^ such as giant planet interiors^6–8^, white and brown dwarfs^9,10^, and the outer layer of neutron stars^11^. On Earth, WDM can nowadays be realized experimentally at large research facilities using different techniques^12^, and particularly advantageous photon properties are offered by x-ray free electron lasers such as LCLS, European XFEL, or SACLA^13–15^. This opens up enticing new possibilities for laboratory astrophysics^16^, the discovery of novel materials^17,18^, and hot-electron chemistry^19^. Consequently, a number of experimental breakthroughs^17,18,20–23^ have been reported over the last years. A particularly important application is given by inertial confinement fusion (ICF)^24,25^, which promises a potential abundance of clean energy in the future. In the currently most well-developed realization, the fuel capsule traverses the WDM regime on its path towards ignition^26^.

Unfortunately, the rigorous diagnosis of such experiments is rendered demanding by the extreme conditions. Indeed, even basic properties such as the temperature, that can be considered as well-known in many other experiments, cannot be directly measured at WDM conditions and have to be inferred from other observations^27^. In this regard, the X-ray Thomson scattering (XRTS) technique^28^ has emerged as a promising method of diagnosis. Yet, the actual inference of the temperature from an experimentally measured XRTS signal is substantially hampered by three major obstacles. Firstly, the theoretical modelling of the dynamic structure factor *S*(**q**, *E*) [with **q** being the wave vector corresponding to the momentum transfer in the scattering process and *E* being the energy shift] of a real WDM system constitutes a challenge^2,3,29^. In practice, one usually has to rely on approximations, such as the Chihara decomposition^27,30^, or time-dependent density functional theory^31^. In addition to their unknown accuracy, state-of-the-art simulations are computationally demanding, which makes them impractical for parameter optimization, and prevents their application to complex materials. Secondly, the experimentally measured XRTS signal is given by the convolution of *S*(**q**, *E*) with the combined source and instrument function (SIF) *R*(*E*): *I*(**q**, *E*) = *S*(**q**, *E*) ⊛ *R*(*E*)^32^. Therefore, important features may be smeared out, and the direct usage of the detailed balance relation on the scattered signal^33^*S*(**q**, − *E*) = *S*(**q**, *E*)*e*^−*βE*^ (with the inverse temperature *β* = 1/*k*_B_*T*) is not always possible. Thirdly, the experimental signal is always afflicted by statistical noise. This may further camouflage physical features, and usually prevents deconvolution.

In this work, we present a complete and straightforward solution to all three obstacles. Specifically, we propose to analyse the two-sided Laplace transform [cf. Eq. (1)] of the measured XRTS signal, ${{{{{{{\mathcal{L}}}}}}}}\left[I({{{{{{{\bf{q}}}}}}}},\, E)\right]$, which gives us direct and unbiased access to the temperature of the probed system in thermodynamic equilibrium. To highlight the flexibility and practical value of our methodology, we apply it to three representative XRTS experiments: (i) the pioneering observation of plasmons in warm dense beryllium by Glenzer et al.^34^; (ii) the study of isochorically heated aluminium by Sperling et al.^35^, which has resulted in an ongoing controversy^36,37^ regarding the nominal temperature of *T* = 6 eV; and iii) a recent XRTS experiment with warm dense graphite by Kraus et al.^27^, where standard interpretation models have resulted in uncertainties of 50% with respect to the temperature. Our method works well in all three cases and, in this way, substantially reduces previous uncertainties.

## Results

### Concept

Let us consider the two-sided Laplace transform of the dynamic structure factor:

1 $${{{{{{{\mathcal{L}}}}}}}}\left[S({{{{{{{\bf{q}}}}}}}},\, E)\right]=\int\nolimits_{-\infty }^{\infty }{{{{{{{\rm{d}}}}}}}}E\,{e}^{-\tau E}S({{{{{{{\bf{q}}}}}}}},\, E)\,.$$

**Fig. 1:**
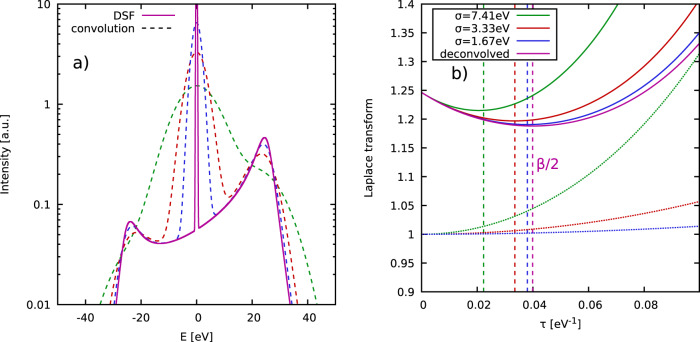
Demonstration of the temperature diagnostics from an XRTS signal. Panel **a** shows synthetic results for the dynamic structure factor (solid purple) in atomic units [a.u.] of a uniform electron gas^29^ (with a narrow Gaussian ion feature around *E* = 0) at the metallic density of ${r}_{s}=\overline{r}/{a}_{{{{{{{{\rm{B}}}}}}}}}=2$ (with $\overline{r}$ being the average electronic separation) computed for the electronic Fermi temperature *T* = 12.53 eV, and for half the Fermi wave number^40^ *q* = 0.5*q*_F_ = 0.91Å^−1^. The dashed curves have been convolved with a Gaussian SIF with the width *σ* = 1.67 eV (blue), *σ* = 3.33 eV (red), and *σ* = 7.41 eV (green). Panel **b** shows the corresponding evaluation of the two-sided Laplace transform of the respective XRTS signals (solid), and SIFs (dotted). Dividing the former by the latter gives the exact curve of *F*(**q**, *τ*) [solid purple] corresponding to the deconvolved dynamic structure factor *S*(**q**, *E*).

In thermodynamic equilibrium, Eq. (1) corresponds to the intermediate scattering function, $F({{{{{{{\bf{q}}}}}}}},\, \tau )\equiv {{{{{{{\mathcal{L}}}}}}}}\left[S({{{{{{{\bf{q}}}}}}}},\, E)\right]$, evaluated at imaginary times *t* = −*iℏτ* ∈ −*iℏ*[0, *β*], which naturally emerges in Feynman’s powerful path integral representation of statistical mechanics^38,39^. *F*(**q**, *τ*) is symmetric around *τ* = *β*/2 [cf. Fig. 1b)]; see the Methods Section. This directly implies that knowledge of *S*(**q**, *E*) gives straightforward access to the actual temperature of the system by solving the simple one-dimensional integral in Eq. (1), and subsequently locating the minimum in *F*(**q**, *τ*) at *β*/2.

An additional obstacle is given by the fact that the XRTS technique does not allow for measurements of *S*(**q**, *E*), but its convolution with the SIF *R*(*E*). While the latter is typically known with high precision, the deconvolution of the measured intensity is generally rendered unstable by the statistical noise. Our concept completely circumvents this obstacle by instead exploiting the convolution theorem of the Laplace transform ${{{{{{{\mathcal{L}}}}}}}}\left[\ldots \, \right]$:

2 $${{{{{{{\mathcal{L}}}}}}}}\left[S({{{{{{{\bf{q}}}}}}}},\, E)\right]=\frac{{{{{{{{\mathcal{L}}}}}}}}\left[S({{{{{{{\bf{q}}}}}}}},\, E) \circledast R(E)\right]}{{{{{{{{\mathcal{L}}}}}}}}\left[R(E)\right]}\,.$$

In practice, we thus compute the two-sided Laplace transform of the experimentally measured intensity, which is very robust with respect to noise. A detailed investigation of the impact of experimental noise onto *F*(**q**, *τ*) is beyond the scope of the present work and will be pursued in future works. The impact of the SIF is then completely removed by the denominator of Eq. (2), which also can be computed in a straightforward way. The accurate determination of the SIF for every shot or experiment should therefore be of paramount importance; indeed it is feasible through source monitoring during the experiment at modern XFEL facilities. As a result, we get the unbiased temperature of any given system from the experimentally measured XRTS signal without the need for theoretical or computational models, and without any bias from the broadening due to the SIF.

### Synthetic data

To demonstrate our methodology, we show synthetic data in Fig. 1. In panel a), we show the XRTS intensity based on a uniform electron gas model^29,40^ (with an additional sharp elastic peak around *E* = 0) at a metallic density (Wigner-Seitz radius ${r}_{s}={\left(3/4\pi {n}_{e}\right)}^{1/3}=2$, with *n*_*e*_ being the electron density; this is close to both beryllium and aluminium) at the electronic Fermi temperature of *T* = 12.53 eV and half the Fermi wave number, i.e., *q* = 0.91Å^−1^. In particular, the solid purple curve shows *S*(**q**, *E*), and the dashed curves have been obtained by convolving the latter with Gaussian instrument functions of different realistic widths, *σ*. With increasing *σ*, the synthetic profiles become broader, and the plasmon peaks around *E* = ±25 eV are smeared out. It is important to note that the convolved curves do not fulfill the aforementioned detailed balance relation between positive and negative energies, so that a direct extraction of the given temperature from such a dataset is not possible.

Let us next consider the corresponding evaluation of the different ingredients to Eq. (2), which are shown in Fig. 1b). The solid purple line corresponds to the actual imaginary-time intermediate scattering function $F({{{{{{{\bf{q}}}}}}}},\, \tau )={{{{{{{\mathcal{L}}}}}}}}\left[S({{{{{{{\bf{q}}}}}}}},\, E)\right]$, which has a minimum at *τ* = *β*/2; see the vertical dashed purple line. Evaluating the two-sided Laplace transform of the convolved curves give the solid blue, red, and green curves, which noticeably deviate from the exact *F*(**q**, *τ*). This is a direct consequence of the broadening due to the SIF *R*(*E*), leading to a violation of detailed balance. Evidently, considering the minimum of the Laplace transform of the convolved signal leads to a substantial overestimation of the temperature (i.e., an underestimation of the inverse temperature *β*), as can be seen particularly well in the case of *σ* = 7.41 eV (vertical dashed green line).

To remove the bias due to *R*(*E*), we have to compute the denominator ${{{{{{{\mathcal{L}}}}}}}}\left[R(E)\right]$ of Eq. (2), which is shown by the respective dotted curves. Indeed, dividing the solid lines by the dotted lines in Fig. 1 b) recovers the true *F*(**q**, *τ*)—and, therefore, the actual value of the temperature *T*—for all cases. We stress that our methodology works over the entire range of wave vectors **q**, including the collective and single-particle regimes. In particular, no explicit resolution of a distinct sharp and narrow plasmon peak in the experimentally measured intensity is required.

### Beryllium experiment

**Fig. 2:**
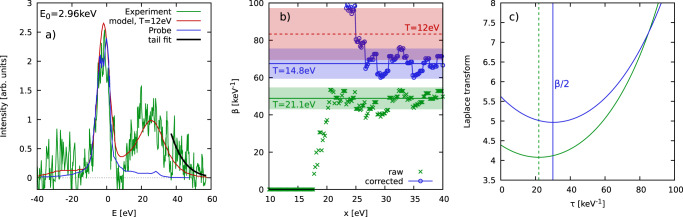
Temperature diagnosis of warm dense beryllium. **a** XRTS measurement by Glenzer et al.^34^ (green), theoretical Mermin model (also taken from Ref. 34) giving *T*_model_ = 12 ± 2 eV (red), SIF *R*(*E*) (blue), and averaged tail for *E* ≥ 40 eV (black); **b** convergence of our model-free temperature diagnosis with respect to the integration boundary *x* of ${{{{{{{\mathcal{L}}}}}}}}\left[S({{{{{{{\bf{q}}}}}}}},\, E)\right]$ with (blue) and without (green) correcting for the instrument function. The dashed line depicts the nominal value of *T*_model_ and has been included as a reference. The shaded areas depict the respective uncertainty range; **c** corresponding results for the imaginary-time intermediate scattering function *F*(**q**, *τ*), with the vertical lines indicating the position of the minimum.

As a first practical application of our diagnostic methodology, we re-examine the pioneering observation of plasmons in warm dense beryllium by Glenzer et al.^34^ in Fig. 2. Panel a) shows the measured XRTS signal (green) together with the instrument function *R*(*E*) (blue) and a theoretical Mermin model^41^ that has been used in Ref. 34 to infer the nominal temperature of *T*_model_ = 12 ± 2 eV. Panel b) shows the temperature as it has been computed from our method both from the convolved signal (green) and by additionally taking into account the instrument function via Eq. (2) (blue). It is important to note that, in actual experiments, one only has the intensity over a finite range of energies, $E\in [{E}_{\min },\, {E}_{\max }]$. We thus truncate the integration boundaries of ${{{{{{{\mathcal{L}}}}}}}}\left[S({{{{{{{\bf{q}}}}}}}},\, E) \circledast \, R(E)\right]$ at ±*x*, and the corresponding results converge around *x* ≳ 30 eV despite the substantial noise in the experimental data.

We extract a temperature of *T* = 14.8 ± 1.5 eV from the experimental data, which is close to the value of *T*_model_ = 12 ± 2 eV (dashed red line and shaded area) that has been inferred from the theoretical Mermin model in Ref. 34. At the same time, we note that the higher temperature from our model-free diagnosis is consistent with the hydrodynamic simulations employed in the original Ref. 34. Moreover, it very plausibly fits to the XRTS signal shown in Fig. 2a), as the red curve noticeably underestimates the averaged tail for *E* ≳ 40 eV (solid black). For completeness, we note that not deconvolving with the SIF would result in the spurious temperature of *T* = 21.1 eV, see the green line in Fig. 2b). Finally, the corresponding results for Eq. (2) with and without the correction due to *R*(*E*) are shown in panel c), which illustrates the robustness of the Laplace transform with respect to noisy input data.

### Aluminium experiment

**Fig. 3:**
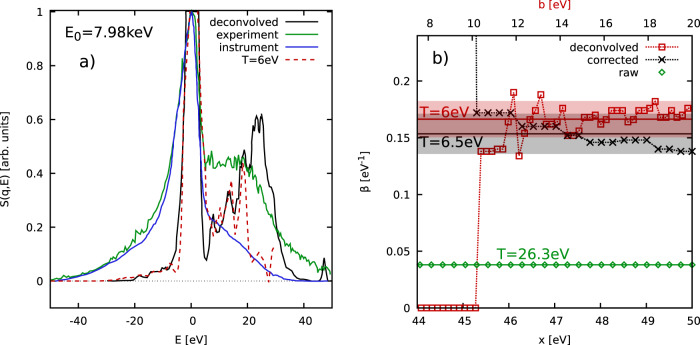
Temperature diagnosis of warm dense aluminium. **a** XRTS measurement by Sperling et al.^35^ (green), deconvolved dynamic structure factor (black), detailed balance estimation of *S*(**q**, *E*) using the nominal value for the temperature of *T* = 6 eV (dashed red), and the SIF (blue); **b** Convergence of the temperature diagnostics with respect to the integration boundary *x* of ${{{{{{{\mathcal{L}}}}}}}}\left[\ldots \,\right]$ of the deconvolved data (red) [with the boundary *b* being shown on the top abscissa], and Eq. ((2)) with (black) and without (green) taking into account the effect of the SIF *R*(*E*). The shaded areas depict the respective uncertainty range.

As a second example, we consider the experiment with isochorically heated aluminium by Sperling et al.^35^ in Fig. 3. This case has the considerable advantage that deconvolved data for *S*(**q**, *E*) are available, see the black curve in panel a); the green and blue curves show the measured XRTS signal and SIF, respectively. In the original publication, Sperling et al.^35^ have found a temperature of *T* = 6 eV based on a detailed balance evaluation of *S*(**q**, *E*). Indeed, the corresponding red curve that has been obtained as *S*_DB_(**q**, *E*) = *S*(**q**, −*E*)*e*^−*E*/6eV^ is in excellent agreement to the deconvolved data in the range of *E* ≲ 20 eV; the final peak around *E* = 30 eV is likely absent from the negative energy range due to its vanishing amplitude in the deconvolved *S*(**q**, *E*). On the other hand, the original interpretation of the XRTS data has subsequently been disputed by independent groups on the basis of time-dependent density functional theory calculations and a model exchange–correlation kernel that has been constructed for the case of a uniform electron gas^36,37^. Specifically, these works have postulated substantially lower temperatures in the range of *T* = 0.3 − 2 eV, and hitherto no decisive conclusion had been reached.

In Fig. 3b), we show the results of our temperature diagnostic as a function of the integration range *x*. Specifically, the black crosses show our evaluation of Eq. (2) taking into account the SIF, and the green diamonds have been obtained without this correction. Evidently, the broadening of the XRTS signal by the SIF plays a decisive role in this data, and leads to a five fold increase in the respective temperature. For the properly corrected data, we find a temperature estimate of *T* = 6.5 ± 0.5 eV, which confirms the previous estimation by Sperling et al.^35^. We also directly compute *F*(**q**, *τ*) from the deconvolved data for *S*(**q**, *E*) via Eq. (1). The results are shown as the red squares, where the upper integration range is denoted as *b* shown on the top abscissa. From panel a), it is clear that the integration only makes sense for ∣*E*∣ ≲ 20 eV, as no significant signal exists in the deconvolved data for *E* < − 20 eV. This analysis gives us a temperature of *T* = 6 ± 0.5 eV, and thereby further substantiates our calculation. Therefore, our present analysis strongly suggests that the temperature estimation in the original Ref. 35 is not an artefact due to the numerical deconvolution, as the direct interpretation of the XRTS signal via Eq. (2) gives the same outcome.

### Graphite experiment

**Fig. 4:**
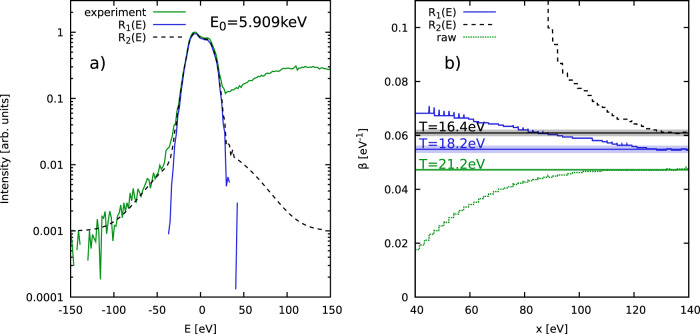
Temperature diagnosis of warm dense graphite. **a** XRTS measurement by Kraus et al.^27^ (green) and possible SIFs *R*_1_(*E*) (blue) and *R*_2_(*E*) (dashed black) shown on a semi-logarithmic scale; **b** Convergence of our model-free temperature diagnosis with respect to the integration boundary *x* with (blue and black) and without (green) taking into account any SIF. The shaded areas depict the respective uncertainty range.

As the final example, we re-examine the recent experiment on warm dense graphite by Kraus et al.^27^ in Fig. 4. In this case, accurate data is available over three orders of magnitude in the measured XRTS signal (green). In addition, the solid blue and dashed black curves show two different models for the SIF. In fact, this uncertainty regarding the true *R*(*E*) has led to an uncertainty in the temperature of ~ 50% based on the applied approximate Chihara models in Ref. 27.

The outcome of our temperature diagnostic is shown in Fig. 4 b). The green curve has been obtained without any correction due to *R*(*E*), leading to the biased temperature of *T* = 21.2 ± 0.2 eV; this is very close to the value of *T* = 21.1 eV given by Kraus et al.^27^ based on the Chihara decomposition. Using the narrow function *R*_1_(*E*) to compute the denominator in Eq. (2) leads to the blue curve, with an estimated temperature of *T*_1_ = 18.2 ± 0.45 eV. Using the broader *R*_2_(*E*) (truncated at *E* ± 90 eV, as the constant asymptotes given in Ref. 27 are clearly unphysical and would lead to a divergent ${{{{{{{\mathcal{L}}}}}}}}\left[{R}_{2}(E)\right]$) for the correction gives the black curve, resulting in a second estimate of *T*_2_ = 16.4 ± 0.35 eV. Evidently, the main source of uncertainty in the interpretation of this experiment is given by the unclear shape of the instrument function, which we estimate by Δ*T* = ±2 eV. In particular, this error bar is an order of magnitude larger than the corresponding uncertainty due to the statistical noise in the intensity. We therefore highlight the importance to accurately determine *R*(*E*) in future experiments. At the same time, we note that our analysis suffers substantially less severely from this drawback compared to the original, Chihara model-based interpretation. Since the existence and precise shapes of the wings in the instrument function are not known, we use the temperature deduced from the narrow function *R*_1_(*E*) as a basis for our final estimate for the temperature, which is given by *T* = 18 ± 2 eV.

## Discussion

In this work, we have presented a highly accurate methodology for the temperature diagnosis of matter at extreme densities and temperatures in thermal equilibrium based on XRTS measurements. In particular, our paradigm does not depend on any model. Therefore, it is free from approximations, and the negligible computation cost makes it highly suitable for the on-the-fly interpretation of XRTS experiments at modern facilities with a high repetition rate, such as the European XFEL^13^. Moreover, it is very robust with respect to the noise of the measured intensity, and completely circumvents the crucial problem of the deconvolution with respect to the combined source and instrument function *R*(*E*). The presented practical application of our technique has given new insights into the behaviour of different materials in the WDM regime, and has substantially reduced previous uncertainties. From a methodological perspective, the present proof-of-principle study opens up the way for the systematic development of our approach into a flexible standard framework for XRTS diagnostics. Future works will include the discussion of spatial gradients, the role of temporal evolution, and the summation over multiple scattering angles contributing from an extended probe volume, which are important e.g. for the diagnosis of fuel capsules in ICF experiments^42^. An additional important item for future investigation is given by the de-facto dependence of the wave vector^43^ on the energy loss, **q** = **q**(*E*), which can likely be neglected for XRTS experiments, but becomes more important for lower energy probe lasers, for example in the context of optical Thomson scattering. Our framework has clear ramifications for the impact of the source and instrument function on the interpretation of the XRTS signal and, in this way, will guide the development of future experimental set-ups.

A key strength of our approach is given by the fact that it is completely model-free and therefore can be straightforwardly applied to arbitrarily complex materials. For example, critical challenges on the path towards achieving high energy gain in ICF implosion experiments are the mitigation of hydrodynamic instabilities and achieving high fuel compression^44^. This requires an improved understanding of radiation transport and hence material opacities along the implosion pathway to improve predictive capabilities of implosion simulations as key information such as the ionization state at high compression are highly controversial^45,46^. This highlights the importance for accurate and robust temperature measurements in complex ablator materials at warm dense matter conditions, which will be enabled by our technique.

Similarly, our idea will boost the burgeoning field of laboratory astrophysics, which is concerned with the study of highly complicated material mixtures at the conditions encountered in planetary interiors^47^.

Finally, we note that XRTS measurements contain a wealth of additional physical information about properties such as the density and the conductivity^48–50^. In this regard, accurate knowledge of the temperature can be used to inform and appropriately constrain existing forward modelling approaches, which, in turn, give access to other parameters. Moreover, we envision the extension of our present framework beyond the temperature, and the direct, model-free extraction of other observables such as quasi-particle excitation energies seems to be promising^39^.

## Methods

### Symmetry of the imaginary-time intermediate scattering function

The symmetry of the imaginary-time intermediate scattering function *F*(**q**, *τ*) directly follows by inserting the detailed balance relation of *S*(**q**, *E*) into Eq. (1) from the main text^39^,

3 $$F({{{{{{{\bf{q}}}}}}}},\, \tau )	=\int\nolimits_{-\infty }^{\infty }{{{{{{{\rm{d}}}}}}}}E\,S({{{{{{{\bf{q}}}}}}}},\, E){e}^{-E\tau }\\ 	=\int\nolimits_{0}^{\infty }{{{{{{{\rm{d}}}}}}}}E\,S({{{{{{{\bf{q}}}}}}}},E)\left\{{e}^{-E\tau }+{e}^{-E(\beta -\tau )}\right\}\\ 	=F({{{{{{{\bf{q}}}}}}}},\, \beta -\tau ).$$

The final relation *F*(**q**, *τ*) = *F*(**q**, *β* − *τ*) can then easily be verified by evaluating the second line of the above equation for $\tau {^\prime}=\beta -\tau$.

## Data Availability

The Laplace transform data, as well as the synthetic spectra shown in Fig. 1, have been deposited in the Rossendorf data repository (RODARE) under accession code https://rodare.hzdr.de/record/2003.
